# Renoprotective Effect of Platelet-Rich Plasma on Cisplatin-Induced Nephrotoxicity in Rats

**DOI:** 10.1155/2018/9658230

**Published:** 2018-07-18

**Authors:** Neveen Salem, Nawal Helmi, Naglaa Assaf

**Affiliations:** ^1^Department of Applied Biochemistry, Faculty of Science, University of Jeddah, Jeddah, Saudi Arabia; ^2^Narcotics, Ergogenic Aids and Poisons Department, Medical Research Division, National Research Centre, Giza, Egypt; ^3^Department of Pharmacology and Toxicology, Faculty of Pharmacy, Misr University for Science and Technology, Cairo, Egypt

## Abstract

Platelet-rich plasma (PRP) has grown as an attractive biologic instrument in regenerative medicine for its powerful healing properties. It is considered as a source of growth factors that may induce tissue repairing and improve fibrosis. This product has proven its efficacy in multiple studies, but its effect on cisplatin-induced nephrotoxicity has not yet been elucidated. The present investigation was performed to estimate the protective impact of platelet-rich plasma against cisplatin- (CP-) evoked nephrotoxicity in male rats. Nephrotoxicity was induced in male Wistar rats by right uninephrectomy followed by CP administration. Uninephrectomized rats were assigned into four groups: (1) control group, (2) PRP group, (3) CP group, and (4) CP + PRP group. PRP was administered by subcapsular renal injection. Renal function, inflammatory cytokines, and growth factor level as well as histopathological investigation were carried out. Treatment with PRP attenuated the severity of CP-induced nephrotoxicity as evidenced by suppressed creatinine, blood urea nitrogen (BUN), and N-acetyl glucosaminidase (NAG) levels. Moreover, PRP depressed intercellular adhesion molecule-1 (ICAM-1), kidney injury molecule-1 (KIM-1), caspase-3, and transforming growth factor-beta 1 (TGF-*β*1) levels, while enhanced the epidermal growth factor (EGF) level. These biochemical results were reinforced by the histopathological investigation, which revealed restoration of normal renal tissue architectures. These findings highlight evidence for the possible protective effects of PRP in a rat model of CP-induced nephrotoxicity, suggesting a new avenue for using PRP to improve the therapeutic index of cisplatin.

## 1. Introduction

Cisplatin or *cis*-diamminedichloroplatinum (II) is one of the most potent antineoplastic agents used to treat a wide assortment of solid tumors, including cancers of the ovaries, testes, head, neck, bladder, cervix, and lung, children's cancers, and some cancers of the blood. It is usually given along with other anticancer drugs [1]. However, nephrotoxicity is a major adverse effect spotted after cisplatin administration. This adverse effect has restricted the clinical use of cisplatin in 25–30% of patients, even after the first dose [2].

Ognjanović et al. [3] demonstrated that cisplatin accumulates in the tubular epithelial cells of the renal proximal tubule, where it is converted to a platinum-glutathione conjugate which is a toxic metabolite and then to a cysteinyl-glycine-platinum conjugate. The latter is further transformed to metabolically reactive thiol a cysteine conjugate that acts as a promoter of cellular kidney injury. The clinical benefits of CP have been restricted due to its nephrotoxic adverse effects [4]. Therefore, creating new agents to alleviate the nephrotoxic effect of cisplatin remains a major goal.

Platelet-rich plasma (PRP) has grown as an attractive biologic instrument in regenerative medicine for its powerful healing properties. PRP is an autologous derivative of whole blood rich in active growth factors. PRP is obtained by centrifuging the blood sample and isolating the platelet-rich supernatant. Then, products such as calcium chloride or fibrinogen are used to activate PRP before application [5]. PRP can include different quantities of plasma, white blood cells, erythrocytes, and platelets according to the device and technique used. The platelet concentration should exceed baseline for whole blood concentration with a minimum fivefold to be considered “platelet rich” [6]. PRP was found to promote tissue regeneration by enhancing cell recruitment, proliferation, and differentiation [7].

Growth factors (GFs) were found to control cell migration, differentiation, proliferation [8], and physiological functions, thereby promotes angiogenesis and tissue regeneration [9]. These GFs include platelet-derived growth factor (PDGF) which promotes type I collagen formation and enhances angiogenesis; transforming growth factor-beta 1 (TGF-*β*1) initiates mesenchymal stem cell proliferation and differentiation and also promotes angiogenesis. Administration of exogenous EGF enhances the regeneration and repair of renal tubule cells and accelerates the restoration of renal function [10]; vascular endothelial growth factor (VEGF) triggers chemotaxis and proliferation of endothelial cells, boosting angiogenesis, vascular hyperpermeability, and renal stem cell differentiation; basic fibroblast growth factor (b-FGF), insulin-like growth factor (IGF), adenosine triphosphate (ATP), angioprotein-2, fibronectin, osteocalcin, and thrombospondin-1 (TSP-1) are among growth factors which are released by PRP [11]. EGF promotes the growth of renal tubular cells that curbs tubular necrosis [12]. IGF is a hormone that alleviates acute tubular necrosis [13]. TGF-*β*1 elevates antiapoptotic Bcl-2 expression, preserves epithelial homeostasis, and prevents renal cell apoptosis [14]. VEGF protects peritubular endothelium, enhances the proliferation of tubular epithelial cells, induces angiogenesis, and promotes renal healing after ischemia [15]. Some studies demonstrated that HGF promotes renal tubular cell regeneration and leads to the repair of kidney structure and function after damage [16]. These growth factors enhance renal tubule cell regeneration, accelerate the recovery of renal function, and repair kidney structure and function after damage [10]. So, it could be anticipated that the administration of PRP as a natural cocktail of GFs to cisplatin-injured kidney would improve its recovery.

Administering growth factors in the form of PRP is better than any other ways as it is a cheap product, easily obtained, and being autologous diminishes the hazards of rejection or immune reaction. Moreover, PRP possesses an antimicrobial action as it contains leukocytes, thus lowering the risk of infection [17].

Despite the fact that PRP has demonstrated to be helpful as a regenerative product as it releases growth factors known to improve tissue damage, its impact on cisplatin-induced renal toxicity has not been previously explored. Therefore, this study was performed to estimate the protective impact of PRP on CP-evoked nephrotoxicity.

## 2. Materials and Methods

### 2.1. Experimental Animals

Fifty adult male Wistar rats (180–220 g) were obtained from the Animal House Colony of the National Research Centre (Cairo, Egypt). The animals were kept in adjusted laboratory conditions (temperature = 25 ± 1°C, humidity = 60 ± 10%, and a 12/12 h light/dark cycle). Animals had free access to standard rat chow and water. Guidelines of the Ethical Committee of National Research Centre, Egypt, were followed, which conform to the recommendations of the National Institutes of Health Guide for Care and Use of Laboratory Animals (publication number 85-23, revised 1996).

### 2.2. Chemicals

Cisplatin (CP) was purchased from Sigma-Aldrich (USA), whereas sodium citrate was from Egyptian International Pharmaceutical Industries Company, Cairo, Egypt.

### 2.3. Preparation of Platelet-Rich Plasma

10 age-matched healthy male Wistar rats were used as PRP donors. The whole blood of rats was drawn through cardiac puncture and mixed with 3.2% sodium citrate at a blood/citrate ratio of 9/1, centrifuged at 400 ×g for 10 minutes, and the supernatant was separated and centrifuged again at 800 ×g for 10 minutes. The top 2/3 which consisted of platelet-poor plasma (PPP) was removed. The remaining layer (1/3) was separated as PRP [18]. PRP was allocated and frozen at −80°C for use. The average PRP was evaluated using a Sysmex XT-1600i system. The platelet count was 2410 × 10^3^ platelets/*μ*L. CaCl_2_ 10% (0.8 mL of PRP + 0.2 mL of CaCl_2_ 10%) was used to activate PRP immediately before its application.

### 2.4. Surgical Procedure

Rats were anesthetized by sodium phenobarbital (50 mg/kg IP) [19]. A right abdominal incision was done; the right renal pedicle was ligated, and a right nephrectomy was performed.

### 2.5. Induction of Nephrotoxicity

Ten days after nephrectomy, renal toxicity was induced in uninephrectomized rats utilizing CP (10 mg/kg) that was administered once intraperitoneally (IP) [20, 21].

### 2.6. Application of PRP in Uninephrectomized Nephrotoxic Rats

24 hours after CP administration, animals were anesthetized by sodium phenobarbital (50 mg/kg IP) and a left abdominal incision was performed. The left kidney was exposed, and activated PRP was directly injected into the kidneys. Five subscapular punctures were performed distributing activated PRP (1 mL) equally over the renal surface. Following the same protocol, other groups were injected with 1 mL of saline and served as saline and positive control groups [22]. It is agreed upon that growth factors are released mainly during the first week after PRP application [11]. Two weeks was set as the end point of the experiment.

### 2.7. Experimental Design

40 adult male uninephrectomized rats were gathered into four groups, 10 rats each: group (1): rats received saline (1 mL, once in the kidney), served as a normal saline control group; group (2): rats received PRP (1 mL, once in the kidney); group (3): rats received CP (10 mg/kg, once IP) to induce nephrotoxicity + saline (1 mL, once in the kidney); and group (4): rats received CP (10 mg/kg, once IP) + PRP (1 mL, once in the kidney).

### 2.8. Sample Collection

After two weeks, blood samples were collected through retroorbital bleeding, centrifuged at 3000 ×g for 15 min (4°C), for serum separation, and stored at −20°C as aliquots for further determinations of renal function: creatinine, blood urea nitrogen (BUN), and N-acetyl-glucosaminidase (NAG). Then, the animals were rapidly decapitated, and the left kidneys of the rats were dissected and rinsed with 0.9% NaCl. Part of the harvested kidneys was homogenized with 0.1 M phosphate-buffered saline at pH 7.4, to give a final concentration of 10% *w*/*v*, and kept at −20°C for the biochemical determinations of intercellular adhesion molecule-1 (ICAM-1), kidney injury molecule-1 (KIM-1), caspase-3, transforming growth factor-beta 1 (TGF-*β*1), and epidermal growth factor (EGF). The other parts of the kidneys were stored in 10% formol-saline at 4°C for subsequent histopathological investigation.

### 2.9. Biochemical Analysis

Serum creatinine concentration was determined kinetically by following the method of [23]. Serum BUN was estimated using the modified Searcey method [24]. Serum NAG was measured according to Luqmani et al. [25]. Renal tissue ICAM-1, KIM-1, EGF (Assaypro, USA), caspase-3, and TGF-*β*1 (Glory Science, USA) were determined by utilizing the methods of solid phase enzyme-linked immunosorbent assay using rat kits according to the manufacturer's instructions.

### 2.10. Histopathological Investigation

Kidney samples were fixed in 10% formalin saline. The specimens were processed and stained with hematoxylin and eosin (H&E), and examined sections (10 fields for each slide) were investigated blindly under the light microscope (Leica, USA) with magnification 400x. The photos were taken by using the AmScope microscopy camera (USA). The renal sections were graded by semiquantitative scale to evaluate the degree of tubular changes. These parameters were evaluated under a 4-point scale: (−) = no alteration, (+) = 10–25% mild altered tubules, (++) = 25 to 50% moderate altered tubules, and (+++) = more than 50% severe altered tubules.

### 2.11. Statistical Analysis

The obtained data were statistically analyzed using SPSS statistical package V. 16 (SPSS Inc., IL, USA). Statistical analysis was performed using one-way analysis of variance (ANOVA) followed by a Tukey post hoc multiple comparison test. Difference was considered significant at *p* ≤ 0.05. Results are shown as median and interquartile range (IQR).

## 3. Results

Three rats died during the experiment and were excluded. Two died after uninephrectomy due to postoperative complications, and the other rat which died after CP administration showed unexpected respiratory distress and reduced mobility.

### 3.1. PRP Ameliorates Kidney Dysfunction and Proximal Tubular Damage in CP Nephrotoxic Rats

**Figure 1:**
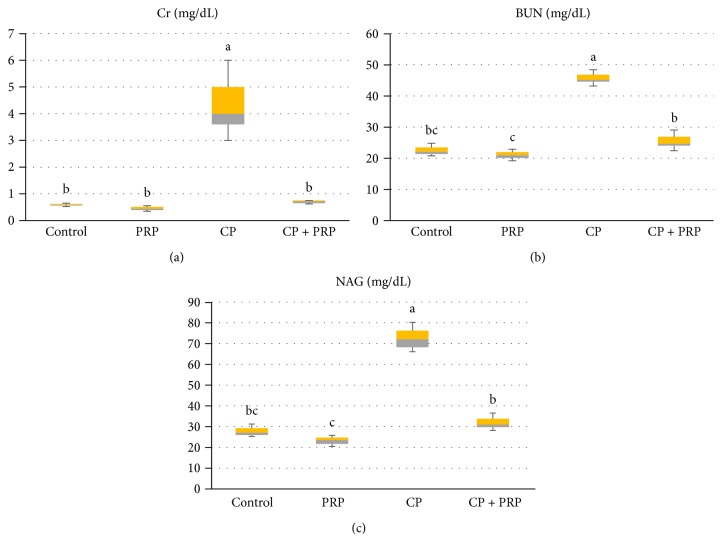
Box and whisker plots showing the effect of PRP administration on serum Cr, BUN, and NAG levels in CP nephrotoxic rats. (a) Creatinine level. (b) Blood urea nitrogen (BUN). (c) N-acetyl-glucosaminidase (NAG). Data are expressed as median and interquartile range. Boxes refer to the 25th (bottom) and 75th (up) percentiles, and the median is the horizontal line inside. PRP: platelet-rich plasma (2410 × 10^3^ platelets/*μ*L); CP: cisplatin (10 mg/kg). Treatments with different letters are significantly different at *p* ≤ 0.05.

CP administration triggered a significant elevation in serum creatinine, BUN levels, and NAG activity (700%, 203 %, and 263%, resp.) versus the control group. PRP administration depressed the levels of the aforementioned parameters (Figure 1). These data indicate the boosting effect of PRP treatment on kidney function and damage.

### 3.2. PRP Alleviates Kidney Injury and Suppresses Apoptotic Markers

**Figure 2:**
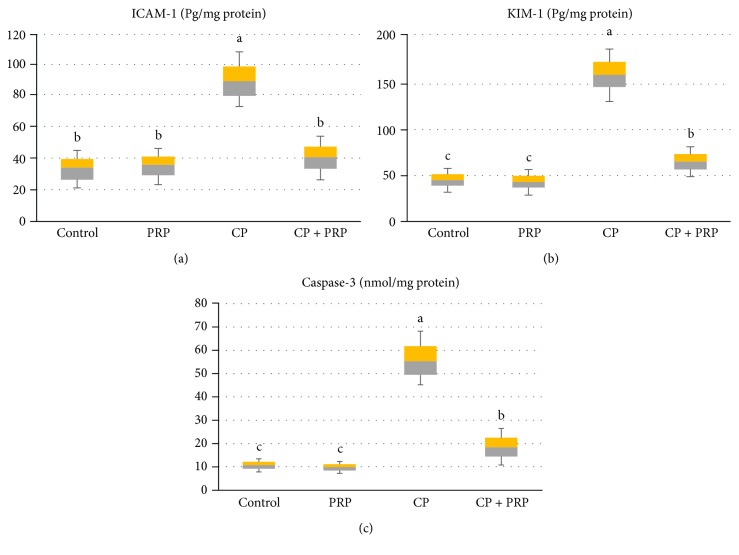
Box and whisker plots showing the effect of PRP administration on renal ICAM-1, KIM-1, and caspase-3 levels in CP nephrotoxic rats. (a) ICAM-1: intercellular adhesion molecule-1. (b) KIM-1: kidney injury molecule-1. (c) Caspase-3. Data are expressed as median and interquartile range. Boxes refer to the 25th (bottom) and 75th (up) percentiles, and the median is the horizontal line inside. PRP: platelet-rich plasma (2410 × 10^3^ platelets/*μ*L); CP: cisplatin (10 mg/kg). Treatments with different letters are significantly different at *p* ≤ 0.05.

CP injection resulted in a significant elevation in renal ICAM-1, KIM-1, and caspase-3 levels by (300%, 310%, and 512%, resp.) as compared to the control group. Meanwhile, PRP treatment counteracted these changes as indicated by significant reduction in these markers (Figure 2). These results suggest that PRP can significantly suppress inflammatory reactions and apoptotic pathway in CP-injected rats.

### 3.3. PRP Downregulates Renal Tissue TGF-*β*1 with Restoration of EGF

**Figure 3:**
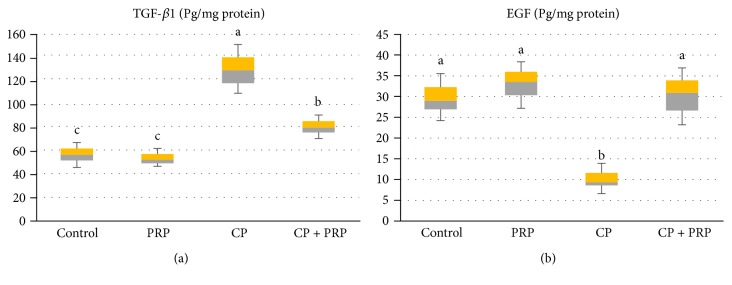
Box and whisker plots showing the effect of PRP administration on renal TGF-*β*1 and EGF levels in CP nephrotoxic rats. (a) TGF-*β*1: transforming growth factor-beta 1; EGF: epidermal growth factor. Data are expressed as median and interquartile range. Boxes refer to the 25th (bottom) and 75th (up) percentiles, and the median is the horizontal line inside. PRP: platelet-rich plasma (2410 × 10^3^ platelets/*μ*L); CP: cisplatin (10 mg/kg). Treatments with different letters are significantly different at *p* ≤ 0.05.

CP significantly elevated the renal TGF-*β*1 level by 2.2 folds, in addition to the significant depression of the EGF level as compared to the control group (Figure 3).

### 3.4. Histological Investigation

**Figure 4:**
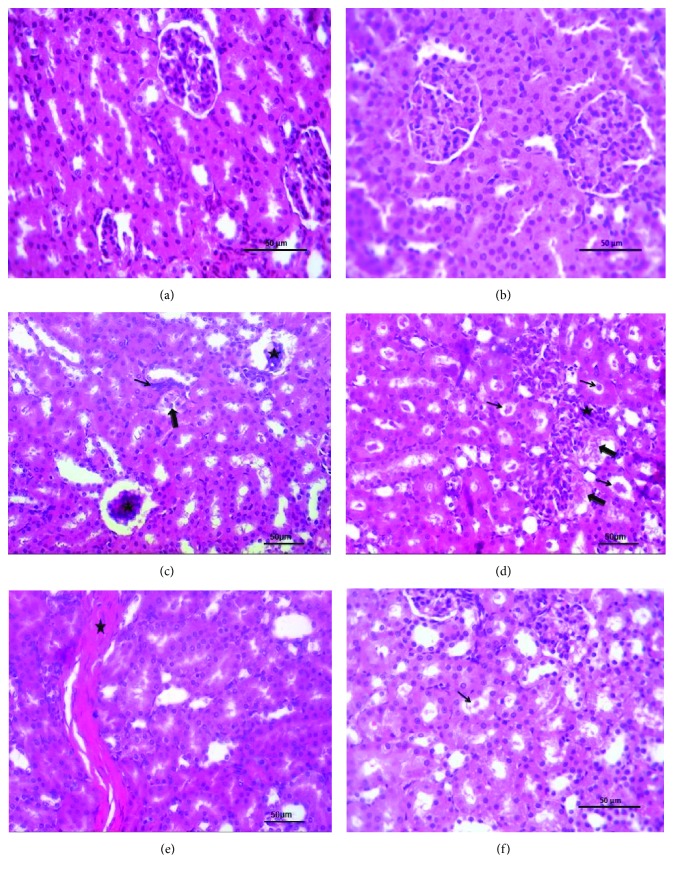
Effect of PRP administration on renal histopathology in CP nephrotoxic rats. Photomicrographs of sections from rat renal tissues. (a) Control group rats received saline showing normal histomorphological architectures. (b) PRP group rats received 1 m PRP elicited no histologic modification. (c, d, e) CP group rats received CP (10 mg/kg) showing (c) necrotic and shrunken glomerular tufts (star), minute peritubular spindle cells (thin arrow) beside sloughed tubular epithelium (thick arrow). (d) Necrotic areas (thick arrow) beside mesangial proliferative glomerulonephritis (star) and numerous casts in injured renal tubule lumina (thin arrow). (e) Interstitial fibrous streaks in corticomedullary junctions (star). (f) CP + PRP group showing a few delicate tubular casts (arrow) within the apparently normal renal tissues. Hematoxylin and eosin staining. Scale bar = 50 *μ*m.

Microscopic examination of rat's kidney sections was scored and represented in Table 1. The examined sections of the control group and PRP group revealed normal structure, normal glomerular, and tubular architecture (Figures 4(a) and 4(b)). While kidney sections of rats treated with CP showed necrotic and shrunken glomeruli (20%), and some glomeruli were lobulated. Moreover, other necrotic glomeruli containing mesangial proliferative glomerulonephritis were observed (Figure 4(c)). Nephritic changes varied from degenerative to necrotic changes in some tubular epithelium besides the fact that heavy casts in the lumina of injured renal tubules were prominent (Figure 4(d)). Endotheliosis and vacuolated media of some renal blood vessels besides interstitial fibrous strands which extend to the neighboring tissue were noticed. Extravasated erythrocytes in some examined sections were seen (Figure 4(e)). Investigation of kidney sections of rat treated with CP + PRP showed that majority of renal parenchyma were apparently normal morphological structure with delicate albuminous casts within some tubules (Figure 4(f)).

## 4. Discussion

The present study investigates for the first time the beneficial impact of PRP on CP-induced nephrotoxicity. CP is a potent and highly effective anticancer agent used nowadays [26]. But its clinical use is limited due to its nephrotoxic side effect [27]. Increasing evidence indicates that oxidative stress, inflammatory cytokines, and apoptosis play some pivotal roles in its pathogenesis [28].

PRP is a powerful therapeutic option for its ability to deliver a great variety of biologically active GFs to the site of injury and is characterized by its simplicity, effectiveness, safety, and constant availability [29]. PRP enhances healing via the secretion of different cytokines and GFs from the alpha granules present in platelets [30]. PRP has an 8-fold increase in GF concentrations as compared to whole blood [31]. So, it could be anticipated that PRP administration as a natural cocktail of GFs with cisplatin would improve kidney recovery.

In the present study, CP administration resulted in impaired glomerular function and renal tubular damage manifested in elevated serum urea and creatinine versus the control group associated with an augmentation in the serum NAG level, which is a proximal epithelium intralysosomal membrane-bound enzyme, released when lysosomal membranes are disrupted. These data agreed with the studies of Saad and Al-Rikabi [32] and Ekor et al. [33]. CP binds to DNA, resulting in the formation of inter- and intrastrand cross-links, hence inhibiting DNA, RNA, protein synthesis, and replication in rapidly proliferating cells. These events enhance tubular damage, especially proximal tubule which receives the highest concentration of cisplatin thereby exacerbating renal insult and leading to renal toxicity, tubular injury, and cell death [34]. Cisplatin interacts with SH groups causing GSH depletion, thereby reducing the cellular antioxidant system and accumulating ROS or its products. Thus, CP initially evokes oxidative renal damage which progresses with a reduced glomerular filtration rate (GFR) and enhances tubular damage followed by morphological architectural deterioration which eventually leads to release of tissue markers in the blood [35]. PRP treatment attenuated renal dysfunction and tubular enzyme leakage as evidenced by suppression of serum creatinine, BUN, and NAG levels via activating intracellular antioxidant enzymes, mainly glutathione peroxidase enzyme (GPx). PRP releases considerable quantities of growth factors (GFs), such as hepatocyte growth factor (HGF), adenosine diphosphate (ADP), adenosine triphosphate (ATP), insulin-like growth factor-1 (IGF-1), and epidermal growth factor (EGF) [16]. These growth factors enhance renal tubule cell regeneration and renal function restoration and repair kidney structure and function after damage [10].

The current data revealed that CP ingestion upregulated inflammatory responses, kidney injury indicators, and apoptotic cascades as evidenced by elevated intercellular adhesion molecule-1 (ICAM-1), kidney injury molecule-1 (KIM-1), and apoptotic marker caspase-3. Overproduction of radicals instigates proinflammatory processes by endothelial cell injury which promotes leukocyte adhesion and infiltration. Generated ROS activates the transcription factor NF-*κ*B, resulting in the synthesis of various proinflammatory adhesion molecules, cytokines, and chemokines such as ICAM-1 and MCP-1 which promote and activate inflammatory cell migration [36] Moreover, kidney injury molecule-1 (KIM-1) is highly expressed in proximal tubular cells following kidney injury as it is considered a specific blood biomarker for acute and chronic kidney injuries [37]. The present results also revealed a significant upregulation in apoptotic markers in the CP-treated renal tissue as indicated by elevation in caspase-3 which belongs to a family of cell death proteases involved in the activation and execution phase of apoptosis. Inflammatory signals together with oxidative stress associated with CP administration have been documented to trigger the upregulation of several genes responsible for cellular death by apoptosis [38]. Interestingly, PRP suppressed renal ICAM-1, KIM-1, and caspase-3 by enhancing the PI3K/Akt pathway which curbs ROS generation, thereby downregulating NF-*κ*B activation and increasing resistance to oxidation [39]. Also, PRP was reported to increase the intracellular expression of the anti-inflammatory mediators (IL-4, IL-10, and IL-13) known to play a major role in inhibiting inflammation and decreasing IL-1*β*-mediated catabolic effect [40]. Moreover, IGF-1 is one of the growth factors in PRP which activates tubular cell regeneration in acute renal failure probably by stimulating the release of growth hormones, which help in tissue repair [41], thus decreases tubular damage and preserves the integrity of the renal parenchyma, glomerular filtration rate, renal blood flow, and renal excretory function [42]. Furthermore, PRP showed antiapoptotic activities via downregulating the expression of apoptotic genes as DAPK1 and BIM mRNA [43] and inhibiting p53, Bax, and caspase-3 levels [44]. Also, HGF in PRP has been shown to interfere in the Fas pathway, thereby rescuing apoptosis in renal cells [45].

Data of the current study demonstrated that CP enhanced the level of renal TGF-*β*1 while lowered the EGF level. These findings coincide with previous studies [46]. The involvement of inflammatory processes has been greatly evidenced in renal injury, including expression of genes that encode proinflammatory cytokines, such as TNF-*α*, interleukin 6 (IL-6), IL-1*β*, and transforming growth factor *β*, which potentiate inflammation [47]. TGF-*β*1 causes renal fibrosis through the production of collagen-rich matrix, starting myofibroblast activation, and epithelial-myofibroblast transdifferentiation [48, 49]. TGF-*β*1 induces apoptosis in renal tubule cells in vitro [50] and in the kidney of transgenic mice in vivo [51]. TGF-*β*1 level has been observed to be augmented in ischemia/reperfusion [52]. Kidney diseases are associated with an alteration in the expression of growth factors and their receptors. For example, the level of EGF was found to be decreased following ischemia and restores its basal level during the recovery phase of ischemia/reperfusion injury [53]. Comparable alterations in the EGF level were observed in patients suffering from acute renal failure [54]. Ledeganck et al. [55] reported that cisplatin resulted in suppression in the epidermal growth factor/epidermal growth factor receptor pathway.

Conversely, PRP administration attenuated TGF-*β*1, while enhanced EGF. PRP possesses powerful mitogenic and chemotactic growth factors involved in initiating the healing process. HGF mediates cellular proliferation, migration, survival, and tissue regeneration. HGF and its receptor c-met are present in the liver, lung, heart, kidney, and brain [56]. HGF possesses a potent antifibrotic ability in the kidney via antagonizing TGF-beta receptor-dependent expression and other profibrotic mediators such as collagen type 1 and fibronectin. Moreover, HGF induced expression of Smad7, an inhibitor of TGF-*β* signaling, in a mitogen-activated protein kinase-dependent manner [57]. HGF prevents activation of interstitial fibroblasts and suppresses tubular epithelial to mesenchymal transition [41]. Epidermal growth factor (EGF) is one of the distinguished growth factors present in PRP and released upon injury. EGF enhances chemotaxis and angiogenesis of endothelial cells and mitosis of mesenchymal cells [58]. Different studies have proven that EGF promotes epithelization and markedly accelerates the healing process. Also, following EGF secretion, cytokine secretion by mesenchymal and epithelial cells is increased. EGF, HGF, and IGF-I boost DNA synthesis in regenerating proximal tubule [59].

Histopathological investigation in this study confirmed the abovementioned biochemical analysis which demonstrated that nephrotic changes varied from degenerative to necrotic changes in some tubular epithelium besides lobulation of glomerular tuft which was common after CP treatment. Moreover, intense albuminous or hyaline casts were detected inside the lumina of the collecting tubules. These findings were confirmed in [60] which revealed CP-induced massive degenerative changes in 50–75% of glomeruli and renal tubules by mechanisms such as oxidative stress and apoptosis. While the CP + PRP-treated group in this study showed that majority of renal parenchyma restore apparently normal renal tissues (accelerated regeneration) with few delicate albuminous casts as compared to the CP group.

Concerning PRP clinical feasibility, PRP therapy is safe given its autologous nature and long-term usage without any reported major complications. For this reason, in addition to its easy availability, it is readily used in clinical and surgical settings such as plastic and maxillofacial surgery, dentistry, and orthopedics [61]. Moreover, it is routinely used in some centers to treat bone fractures, as an aid in dental implants and prosthesis, and to treat diabetic ulcers and dry eye in Sjögren's syndrome [62]. However, limitation in evaluating the clinical effects of PRP is variation in established preparation protocols. An additional variation in the PRP product results from patient differences in age, medical comorbidities, and healing capabilities [63]. Continued basic science research elucidating the downstream effects of PRP can help drive clinical research and develop clinical recommendations for the use of PRP.

## 5. Conclusions

Our findings highlight evidences for the protective effects of PRP in a rat model of CP-induced nephrotoxicity. These effects were mediated through suppressing inflammatory mediators, boosting renal antioxidant defense, curbing apoptosis, accelerating the recovery of renal function, and repairing kidney structures after damage. Collectively, this study could open a new avenue for using PRP to improve the therapeutic index of cisplatin.

## Figures and Tables

**Table 1:** Semi-quantitative scoring for renal injury in different experimental groups.

Lesion	Control	PRP	CP	CP + PRP
Necrosis of glomerular tufts	−	−	+++	−
Necrosis of renal tubules	−	−	++	−
Acute cell selling of epithelial renal tubule	−	−	+++	+
Congestion of renal blood vessels	−	−	++	−
Hemorrhages	−	−	+	−
Fibrosis	−	−	++	−
Tubular casts	−	−	+++	++

− = no alteration; + = 10–25% mild altered tubules; ++ = 25 to 50% moderate altered tubules; +++ = more than 50% severe altered tubules.

## Data Availability

The data used to support the findings of this study are available from the corresponding author upon request.

## Additional Points

*Highlights*. (i) Platelet-rich plasma protects against cisplatin-induced nephrotoxicity in a rat model. (ii) PRP suppresses inflammatory mediators, boosts renal antioxidant defense, and curbs apoptosis. (iii) Also, it accelerates the recovery of renal function and repairs kidney structure after damage induced by cisplatin.
